# Antimicrobial Properties of Copper Nanoparticles and Amino Acid Chelated Copper Nanoparticles Produced by Using a Soya Extract

**DOI:** 10.1155/2017/1064918

**Published:** 2017-02-13

**Authors:** I. DeAlba-Montero, Jesús Guajardo-Pacheco, Elpidio Morales-Sánchez, Rene Araujo-Martínez, G. M. Loredo-Becerra, Gabriel-Alejandro Martínez-Castañón, Facundo Ruiz, M. E. Compeán Jasso

**Affiliations:** ^1^Doctorado Institucional en Ingeniería y Ciencia de Materiales, Universidad Autónoma de San Luis Potosí, San Luis Potosí, SLP, Mexico; ^2^Facultad de Ciencias, Universidad Autónoma de San Luis Potosí, San Luis Potosí, SLP, Mexico; ^3^Facultad de Estomatología, Universidad Autónoma de San Luis Potosí, San Luis Potosí, SLP, Mexico; ^4^Departamento Físico Matemático, Universidad Autónoma de San Luis Potosí, San Luis Potosí, SLP, Mexico; ^5^Coordinación para la Innovación y Aplicación de la Ciencia y Tecnología, Universidad Autónoma de San Luis Potosí, San Luis Potosí, SLP, Mexico

## Abstract

This paper reports a comparison of the antibacterial properties of copper-amino acids chelates and copper nanoparticles against* Escherichia coli*,* Staphylococcus aureus*, and* Enterococcus faecalis*. These copper-amino acids chelates were synthesized by using a soybean aqueous extract and copper nanoparticles were produced using as a starting material the copper-amino acids chelates species. The antibacterial activity of the samples was evaluated by using the standard microdilution method (CLSI M100-S25 January 2015). In the antibacterial activity assays copper ions and copper-EDTA chelates were included as references, so that copper-amino acids chelates can be particularly suitable for acting as an antibacterial agent, so they are excellent candidates for specific applications. Additionally, to confirm the antimicrobial mechanism on bacterial cells, MTT assay (3-[4,5-dimethylthiazol-2-yl]-2,5-diphenyltetrazolium bromide) was carried out. A significant enhanced antimicrobial activity and a specific strain were found for copper chelates over* E. faecalis*. Its results would eventually lead to better utilization of copper-amino acids chelate for specific application where copper nanoparticles can be not used.

## 1. Introduction

It is well known that the copper has a wide activity against bacteria and fungi [1–4]. Due to the development of antibiotic resistance and the surging of infectious diseases, a lot of researchers are searching for new antibacterial agents [5–11].

Although copper is one of the most widely used materials in various applications, its nanosynthesis requires some special care because of their high holding to rust. Compared to gold and silver, copper is extremely sensitive to air, and copper oxides phases are thermodynamically more stable. Therefore, the formation of an oxide layer on the surface of the copper nanoparticles is inevitably causing a marked decrease of its antibacterial properties [12–14].

In the last few years, several organics copper complexes of Ni, Cu, and Co have been tested for their antibacterial and antitumor properties. Their mode of action is probably the binding of amino acids and metals ions, which is likely to leave some potential donors of free atoms and enhance the biological activity [13, 15–17].

Chelates are highly stable products capable of maintaining the surrounded metal ions from an organic molecule (chelating agent); so its precipitation as insoluble hydroxides should be avoided with the care of the storage [15, 18].

We have previously reported about a soybean aqueous extract mediated synthesis of very stable copper nanoparticles; in this work we report the antibacterial activity of copper nanoparticles and copper chelated species that are an intermediate step between ion copper and copper nanoparticles [19]. The main objective was to compare the antibacterial properties of different species of copper, such as copper nanoparticles and copper chelates. Such comparative study would reveal strain specificities and would eventually lead to better utilization of copper-amino acids chelate for specific application where copper nanoparticles could be not used.

## 2. Materials and Methods

### 2.1. Reagents

Textured soy was purchased in a local natural products supermarket; sodium hydroxide (NaOH) and copper sulfate (CuSO_4_·5H_2_O) of Sigma-Aldrich were used without any further purification. Milli-Q water (18.2 Ω) was used throughout the experiment; and the synthesis reagents are phosphate buffer Na_2_HPO_4_ and KH_2_PO_4_ (Fermont), Mueller-Hinton broth (BD Difco), sodium chloride (NaCl, CTR Scientific), and Kit MTT 3-[4,5-dimethylthiazol-2-yl]-2,5-diphenyl tetrazolium bromide (Sigma-Aldrich).

### 2.2. Characterization

Optical absorption spectra were obtained with an Ocean Optics S2000-UV-Vis system, Transmission Electron Microscopy (TEM) images at 100 kV were carried out using a JEOL-1230, Thermogravimetric Analysis (TGA) was made, and the average particle size was measured with a Dynamic Light Scattering Nanosizer (DLS). Infrared spectroscopy (FTIR) was made with an IR Affinity-1.

### 2.3. Experimental Method

Six samples were prepared to compare the bactericidal activity; the samples with copper were synthesized with a concentration of 40 mM. The syntheses are described as follows:

- Soybeans extract: the solution was prepared by heating to boiling point 100 mL of deionized water with 3 g of textured soya for 10 min; the fiber was then separated by filtration.
- Ionic solution of Cu^2+^ species: this solution was prepared adding CuSO_4_ in deionized water.
- Copper-soybean extract chelates: they were synthetized according to the method previously reported by Guajardo-Pacheco et al. [19]. A soybean extract solution was prepared, as described above; after that, 1 gr of CuSO_4_·5H_2_O was added and mixed for 15 min in magnetic stirring; the pH was adjusted to 7 using a 1 M solution of sodium hydroxide.
- Colloidal dispersion of copper nanoparticles: the particles were prepared in the aqueous phase by a chemical reduction of copper chelate complex solution using sodium borohydride as reducer. 100 mL solutions of copper chelate complex were purged with N_2_ gas for 10 min to remove the dissolved oxygen; 1 mL of aqueous solution of sodium borohydride (0.1 M) was then added to it drop-wise under constant stirring. The solution turned into a black color on the complete addition of the reducing agent, denoting the formation of copper nanoparticles. The nanoparticles were washed repeatedly with ethylic alcohol [19].
- EDTA-copper chelate: for EDTA chelated copper solution, 1 gr of CuSO_4_·5H_2_O and 1 gr of EDTA were mixed in 100 mL of deionized water. The Cu/EDTA ratio was calculated previously by 11.68.

### 2.4. Antimicrobial Test

The antimicrobial activity of the chelates was tested using the standard microdilution method, which determines the minimum inhibitory concentration (MIC) and the minimum bactericide concentration (MBC), leading to the inhibition of bacterial growth (CLSI M100-S25 January 2015). The strains tested were* Escherichia coli* (ATCC 25922),* Staphylococcus aureus* (ATCC 29213), and* Enterococcus faecalis* (ATCC 29212). The bacterial concentration was standardized to an optical density of 0.2 at 568 nm (approximately 1 × 10^8^ CFU/mL) using the McFarland scale. The chelates in dispersion were diluted with 50 *μ*L of Mueller-Hinton broth and 50 *μ*L of phosphate buffer inoculated with the tested strains at a concentration of 1 × 10^5^ CFU/mL was grown for 24 h at 35 ± 1°C [20].

### 2.5. MTT Assay

The MTT assay was used for measuring the proliferation of bacterial cells. Bacterial cells (10^5^ CFU/mL) were incubated into Mueller-Hinton broth at 50 *μ*L per well in 96-well microtiter plates. Threefold serial dilutions of copper-amino acids chelate were added to wells containing bacterial cells. After 24 h of incubation at 37°C, each concentration was tested in triplicate. 24 hours later, 10 *μ*L of the MTT (5 *μ*L/mL) reagent was added to each well and the plates were incubated for 4 h at 37°C. Then, DMSO (100 *μ*L) was added to finish the reaction; the plate was shaken gently to redissolve the crystals formed. The absorbance was read at 490 nm wavelength by a Microplate Reader (Bio-Rad Laboratories) spectrophotometer. The results were expressed as the inhibition ratio of cell increase calculated as [(*A* − *B*)/*A*]*∗*100%, in which *A* and *B* represent the average of live bacteria of the control and samples, respectively [21].

## 3. Results and Discussion

**Figure 1:**
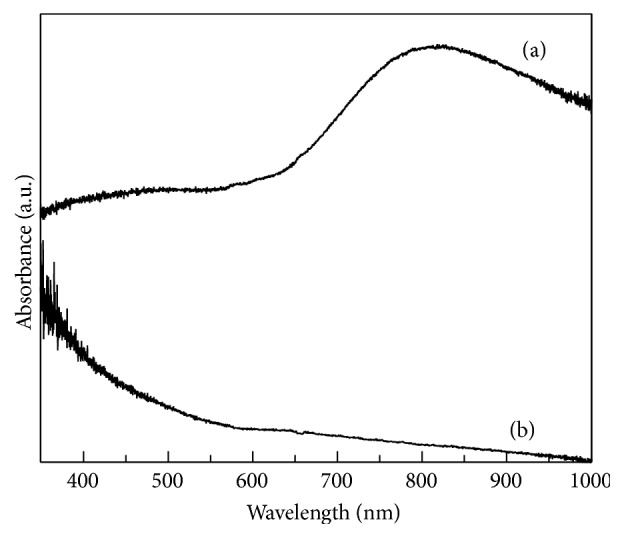
UV-Vis spectrum of (a) ionic solution of copper and (b) copper-amino acids chelate.

Figure 1(a) shows UV-Vis absorption spectra of the copper ions solution and Figure 1(b) copper-soybeans extract solution. It is possible to observe the typical wide band associated to copper free ions in the range between 650 and 1000 nm with a maximum at approximately 800 nm [22]. In Figure 1(b) this band disappears indicating the depletion of the copper free ions and formation of a copper-soya extract complex. The band in the range between 400 and 550 nm has been ascribed to soybeans extract.

**Figure 2:**
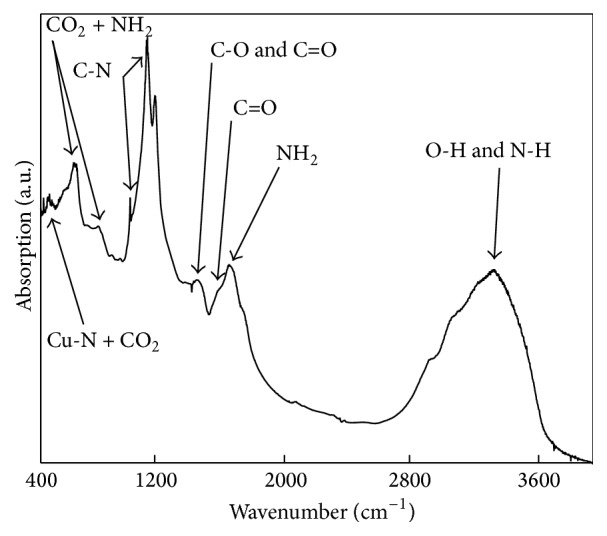
Infrared absorption spectra of copper-amino acids chelate.

Figure 2 shows the absorption infrared spectrum of the copper-soybean extract solution. There can be seen bands in the range from 400 to 500 cm^−1^ corresponding to the formation of the copper chelate, the band at 457 cm^−1^ is assigned to the contribution of the Cu-N symmetric vibration and CO_2_ wagging, and the bands at 614 cm^−1^ and 773 cm^−1^ can be ascribed to the contribution of CO_2_ rocking and NH_2_ rocking due to complex with amino acids; from 1000 to 1100 cm^−1^ are the C-N vibrations. The peaks of *ν* (C-O) and *ν* (C=O) at 1421 cm^−1^, 1580 cm^−1^, and 1626 cm^−1^ are the contribution of *ν* (C=O) and NH_2_ scissoring, respectively. The range from 3290 to 3390 cm^−1^ corresponds to O-H and N-H vibrations. All of these vibrations modes confirm the copper-amino acid complex formation [23]; it is well known that the soybean is rich in proteins content, of which the 95% of the total protein is called soluble protein, and approximately 16% is attributed to the Nitrogen presence [24, 25], favoring the copper-amino acids chelates formation as can be seen in the infrared absorption spectra.

**Figure 3:**
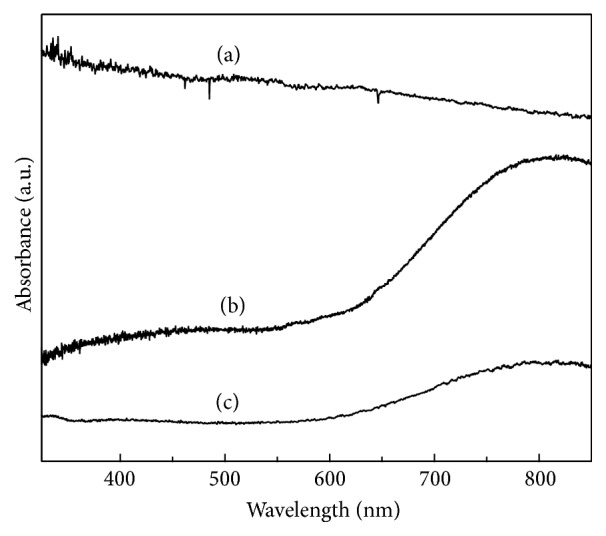
UV-Vis spectrum of (a) EDTA solution, (b) ionic solution of copper, and (c) EDTA-copper chelate.

Figure 3 shows the UV-Vis absorption spectrum of (a) EDTA starting solution, where a wide band in the range of 400 to 900 nm can be observed, and (b) ionic copper solution with its typical band wide range between 400 and 900 nm and the maximum being approximately at 800 nm. And (c) corresponds to the EDTA-copper chelate sample; as it is possible to see, the maximum of ionic copper band decreases due to the copper free ions depletion and formation of copper chelate with the EDTA; this might represent a proof of chelate formation.

**Figure 4:**
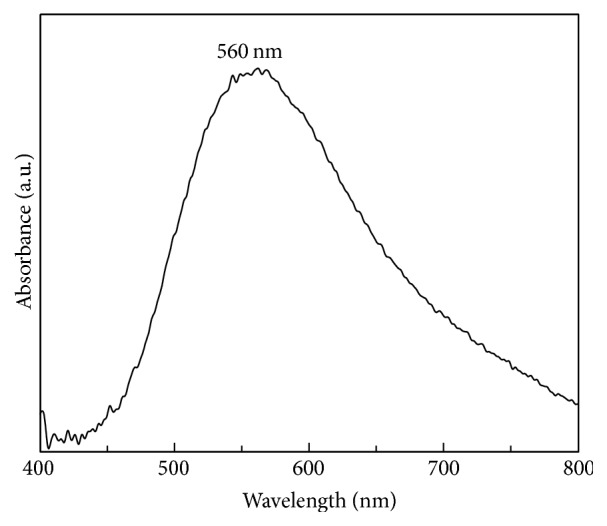
UV-Vis of copper nanoparticles sample.

Figure 4 shows the UV-Vis absorption spectrum of the copper nanoparticles sample with a typical absorption plasmon band at approximately 560 nm.

**Figure 5:**
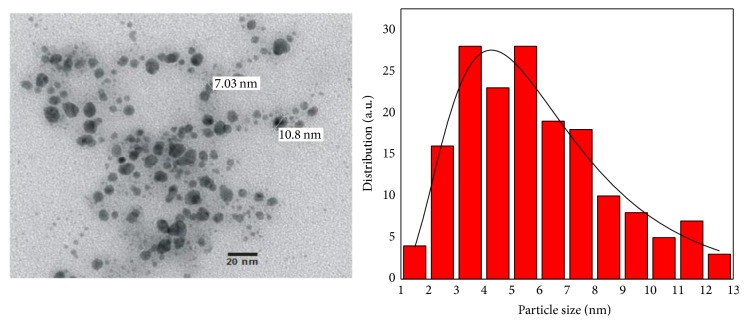
TEM image of copper nanoparticles sample and the particle size distribution histogram.

**Figure 6:**
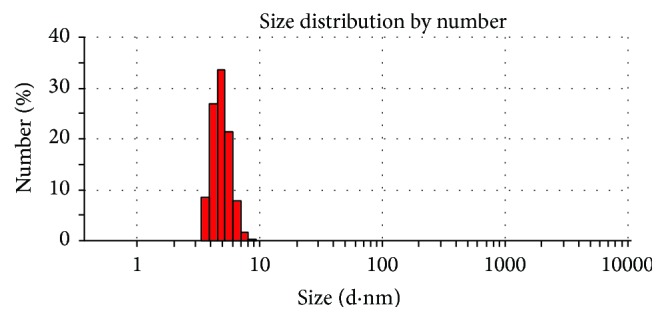
Dynamic Light Scattering measurement of copper nanoparticles sample.

Figure 5 shows a TEM image of copper nanoparticles sample, in which there can be observed that the nanoparticles have quasi-spherical shapes with an average diameter of 5.67 ± 0.5337 nm as it can be seen in the histogram, where 169 nanoparticles were measured. The TEM image and the histogram also show a narrow size distribution of the nanoparticles; this could be corroborated by Dynamic Light Scattering measurements (Figure 6).

Figure 6 shows the Dynamic Light Scattering measurement of copper nanoparticles; the average size obtained was of 4.9 nm with a narrow size distribution.

### 3.1. Antibacterial Results

MIC values were obtained for the chelates tested against* E. coli* (ATCC 25922),* S. aureus* (ATCC 29213), and* E. faecalis* (ATCC 29212). The results are presented in Table 1, where it can be seen that the copper-amino acids chelate sample presents the best antibacterial activity against the three strains tested in comparison with copper ions, copper-EDTA chelate, and copper nanoparticles.

The MIC of the copper nanoparticles and the copper-amino acids chelates is lower when it is tested against* E. faecalis* than when tested against* E. coli* and* S. aureus*. These results may refer to differences in the cell wall of each strain; the cell wall of Gram-positive strains is wider than the cell wall of Gram-negative strains [26]. In addition, the reported mechanism for the toxicity of copper ions on* E. faecalis* includes a rapid DNA degradation, followed by a reduction of bacterial respiration; it is also known that copper ions inhibit certain cytochromes in the membrane of* E. faecalis* by altering the conformation and electron transference of associated reductases [27]. These affectations of copper over* E. faecalis *could be potentiated with the presence of amino acids in the copper chelate and this could be the reason why* E. faecalis* is more sensitive to copper-amino acids chelate than* E. coli* and* S. aureus*.

As for the copper nanoparticles and the EDTA reference there is no significant difference between the MIC results of* E. coli* and* S. aureus*.

**Figure 7:**
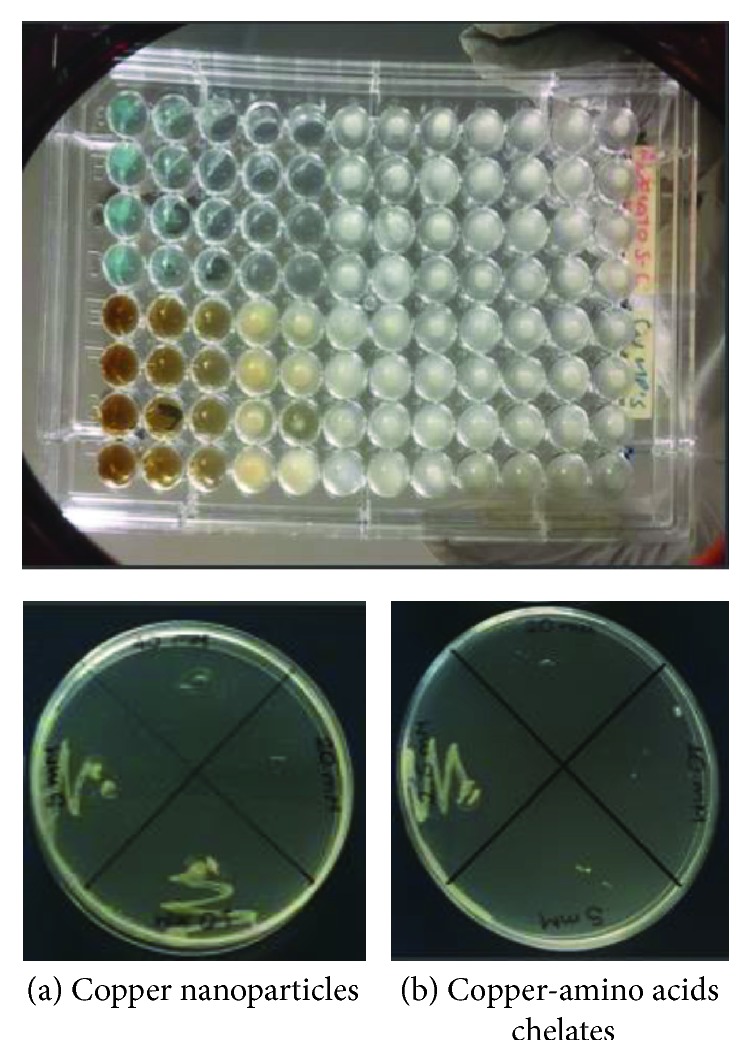
MICs of nanoparticles against bacterial strain* Enterococcus faecalis*. (a) Copper nanoparticles; (b) copper-amino acids chelate.

Figure 7 shows the MICs of copper nanoparticles and copper-amino acids chelate against bacterial strains* E. faecalis*. The serial dilutions of nanoparticles were exposed to bacterial strains, and we can see that the copper-amino acids chelate antimicrobial effect is better than that of the copper nanoparticles; in general, the copper-amino acids chelate showed the highest activity among all the six samples in the three bacteria tested (Table 1).

**Figure 8:**
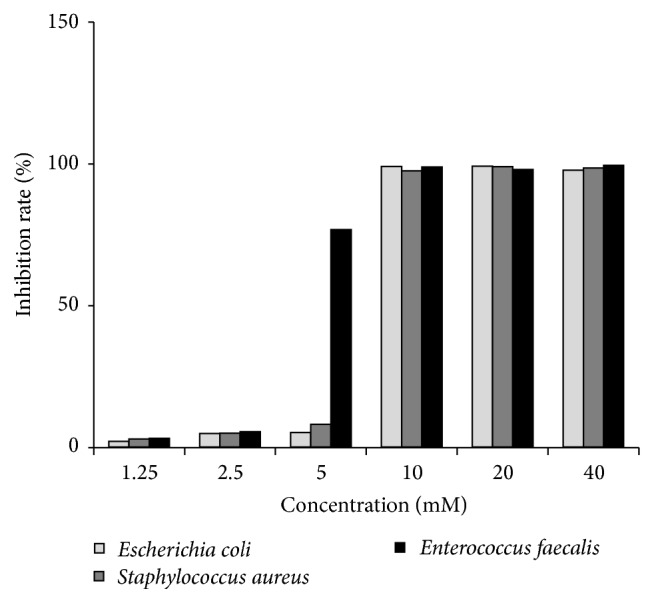
Effect of copper-amino acids chelate sample by MTT assay.

In Figure 8, we can see MTT assay results for the copper-amino acids chelate sample; the Inhibition Rate value for* E. faecalis* is higher compared to* E. coli* and* S. aureus* at a concentration of 10 mM, representing one order of magnitude smaller compared to the concentrations obtained for the other tested solutions.

## 4. Conclusions

Copper-amino acids chelate was synthetized in aqueous solution and antimicrobial assays, which demonstrates that its strain specificities and enhanced activity are about ten times more effective comparing to the copper nanoparticles and the copper-EDTA chelate, obtaining a better antimicrobial effect against* E. faecalis* rather than both* E. coli* and* S. aureus*. These results show that copper-soy chelates can be particularly suitable as antibacterial agent candidates for specific application instead of copper nanoparticles.

## Figures and Tables

**Table 1:** Minimum inhibitory concentrations (MIC).

Sample	MIC copper (mM)		
	Bacterial strains		
	*E. coli* (ATCC 25922)	*S. aureus* (ATCC 29213)	*E. faecalis* (ATCC 29212)
Soybeans extract	—^a^	—^a^	—^a^
Ionic solution of Cu	20 ± 0	20 ± 0	20 ± 0
Copper-amino acids chelate	10 ± 0	10 ± 0	5 ± 0
Copper nanoparticles	40 ± 0	40 ± 0	20 ± 0
EDTA-copper chelate	20 ± 0	20 ± 0	20 ± 0
EDTA solution	40 ± 0	40 ± 0	40 ± 0

^a^The antibacterial activity was not found with the mM concentrations used in this work.
